# Metabolomics and transcriptomics indicate the changes in medicinal components of *Amygdalus mongolica* kernels during different developmental stages

**DOI:** 10.3389/fpls.2025.1597638

**Published:** 2025-05-23

**Authors:** Zhenyu Lei, Hong Chang, Li Song, Hongbing Zhou, Wanfu Bai, Yingchun Bai, Shuyuan Jiang, Songli Shi, Jia Wang

**Affiliations:** ^1^ Department of Pharmacy, Baotou Medical College, Inner Mongolia University of Science and Technology, Baotou, China; ^2^ Institute of Bioactive Substance and Function of Chinese Materia Medica and Mongolian Medicine, Baotou Medical College, Inner Mongolia University of Science and Technology, Baotou, China

**Keywords:** *Amygdalus mongolica*, developmental phases, amino acids, phenylalanine metabolic, bioanalytics

## Abstract

*Amygdalus mongolica* is a traditional Chinese and Mongolian medicine and is quite effective in relieving cough and eliminating phlegm. In this study, ultra-high performance liquid chromatography-mass spectrometry (UPLC-MS) was used to perform metabolomics analysis, combined with transcriptomic sequencing, to study the samples of *A. mongolica* at early, middle and late growth stages. A comprehensive analysis was conducted to systematically investigate the dynamic changes and developmental trajectories of kernel progression across distinct growth stages, utilizing differential expression analysis and pathway enrichment approaches. A total of 104 significant differential metabolites were identified across different growth stages: the early growth stage (E), the middle growth stage (M), and the late growth stage (L). Specifically, 68 and 36 differential metabolites were detected in the comparisons of E vs. M and M vs. L, respectively. Among them, 29 and 21 metabolites exhibiting up-regulation. In comparison, 39 and 15 metabolites demonstrated down-regulation. Transcriptomic analysis identified 133 differentially expressed genes associated with 62 pathways in the E vs. M. Simultaneously, 14 differentially expressed genes linked to 13 pathways in the M vs. L. The accumulation of various metabolites varied across the growth stages, with a marked increase in amino acids during the middle and late developmental phases, alongside fluctuations in the levels of flavonoid compounds. In conclusion, the study reveals significant variations in the content and types of amino acid compounds and the expression levels of key genes within relevant pathways of *A. mongolica* kernels throughout different growth stages. Especially, the increase in flavonoid compound expression with advancing growth stages highlights their substantial medicinal potential. These findings provide a foundation for future developing and utilizing valuable medicinal ingredients in *A. mongolica* kernels.

## 1 Introduction

*Amygdalus mongolica* (Maxim.) Ricker, a deciduous shrub belonging to the genus *Amygdalus* within the Rosaceae family, is recognized for its significant drought tolerance. This species is characterized by a robust root system that enables it to withstand both drought and cold conditions. It can thrive in soils with low nutrient availability. *A. mongolica* is native to the Mongolian Plateau and the Alxa Desert, primarily in western Inner Mongolia, Gansu, and Ningxia (Zhao, 1995). The *A. mongolica*, a shrub species characterized by its strong environmental adaptability, robust root system, and vigorous growth, predominantly flourishes on rocky mountain slopes. The flowering period of *A. mongolica* is usually in mid-April. Generally, the fruit fully develops and matures 120 days after flowering and pollination in August (An and Fang, 2009). Upon maturation, the outer and middle layers of the fruit’s skin rupture, resulting in the dispersal of seeds that subsequently settle in the soil of gentle inclines or within rock crevices. Under favorable environmental conditions, these seeds germinate, leading to the establishment of seedlings (Wang et al., 2015). This classification framework, grounded in morphological and physiological transformations, holds substantial value for investigating variations in metabolic profiles (Zhang et al., 2025). It not only enhances our comprehension of the dynamic evolution of metabolism but also establishes a robust theoretical basis for identifying key metabolites and metabolic pathways, as well as for informing resource utilization and varietal breeding strategies.

The ecological significance of the *A. mongolica* is notable, as it plays a crucial role in mitigating wind erosion and promoting water and soil conservation. Furthermore, its medicinal properties are recognized for their efficacy in alleviating constipation by moistening the intestines and addressing phlegm and cough. Additionally, the branches and leaves of the *A. mongolica* are abundant in amino acids and proteins essential for animal nutrition, thereby possessing considerable feed value (Zhang et al., 2022). As a traditional medicinal herb in both Chinese and Mongolian cultures, *A. mongolica* has been found to contain various biologically active compounds, including proteins (Zhou et al., 2015), flavonoids (Shi et al., 2013), fatty acids and unsaturated fatty acids (Zhu, 2013), organic acids (Zheng et al., 2013), polysaccharides (Shi et al., 2011), amygdalin (Bai et al., 2015), and amino acids (Wang et al., 2012), all of which contribute to its nutritional and therapeutic properties. Research has demonstrated that extracts from *A. mongolica* exhibit therapeutic effects against conditions such as hyperlipidemia (Zheng et al., 2017), renal fibrosis (Gao et al., 2022), liver fibrosis (Wu et al., 2021), and pulmonary fibrosis (Quan et al., 2020), highlighting the pharmacological potential of this species for further investigation and development. Plant growth is a complex biological process characterized by dynamic changes in nutrient composition and the decomposition and synthesis of various substances that support normal development. Given its importance as a traditional medicinal herb in both Chinese and Mongolian cultures, the sustainable protection and utilization of *A. mongolica* have become critical areas of research. While existing studies primarily focus on the pharmacological properties of *A. mongolica*, there is a notable lack of research regarding the metabolite accumulation patterns during this species’ fruit development.

Metabolomics represents a rapidly evolving domain within systems biology, extensively employed in the investigation of plant growth through the comprehensive analysis of small molecular metabolites present in biological samples (Gao and Zhao, 2024). The research about plant metabolites is progressing at a swift pace. The utilization of high-throughput analytical methodologies, such as MS and NMR, enables researchers to conduct more thorough analyses and identifications of plant metabolites (Hao et al., 2025). Transcriptomics, a fundamental component of systems biology research, serves as a conduit that links the static sequence information of the genome with the dynamic functional expression of the proteome by examining the spatiotemporal expression patterns of all transcripts within an organism. RNA not only fulfills the role of conveying genetic information via transcription but also acts as a central medium within the multilayered regulatory network governing gene expression (Athanasopoulou et al., 2021; Rurek and Smolibowski, 2024). The integration of metabolomics and transcriptomics research facilitates the exploration of the relationship between metabolites and genes, thereby elucidating alterations in phenotypic expression (Hager et al., 2025; Mesnage et al., 2018). This study aims to utilize metabolomic techniques to analyze the diversity and abundance of secondary metabolites within metabolic pathways, while also employing differentially expressed genes identified through transcriptomic analysis to elucidate the regulatory mechanisms that govern metabolite biosynthesis in medicinal plants.

## 2 Materials and methods

### 2.1 Plant materials

The experimental materials used in this study were grown in Jiufeng Mountain in Baotou City, Inner Mongolia. The kernel of *A. mongolica* was harvested at three different developmental stages: 60 days after pollination (early development stage, E), 90 days (middle development, M), and 120 days (late development, L), as illustrated in **Supplementary Figure S1**. At each developmental stage, three biological replicates were collected. The kernels were meticulously excised from the fruit and immediately flash-frozen in liquid nitrogen, then stored at -80°C for subsequent analyses.

### 2.2 Metabolite extraction and information analysis

Kernel samples of *A. mongolica* were thawed and subjected to metabolite extraction using a 50% methanol. Specifically, a 20 µl aliquot of the sample was mixed with 120 µl of pre-cooled 50% methanol, vortexed for one minute, and then incubated at room temperature for ten minutes. The resulting extraction mixture was stored at -20°C overnight. The mixture was centrifuged at 4000g for 20 minutes, and the supernatant was transferred to a new 96-well plate. All chromatographic separations were performed using a UPLC system, specifically an ACQUITY UPLC BEH Amide column, with the column oven maintained at 35°C. The flow rate was set at 0.4 ml/min, and the mobile phase consisted of solvent A (25 mM ammonium acetate + 25 mM NH_4_H_2_O) and solvent B (isopropanol: acetonitrile = 9:1 + 0.1% formic acid), with an injection volume of 4 µl per sample. The metabolites eluting from the column were detected using a high-resolution tandem mass spectrometer, the TripleTOF 5600 plus, which operated in both positive and negative ion modes. The chromatographic and mass spectrometric processes were managed using XCMS software version 3.2.0.

The instrumentation employed included a high-performance liquid chromatograph (model Thermo Scientific UltiMate 3000 HPLC, Thermo Scientific) and the high-resolution mass spectrometer, TripleTOF 5600 plus (SCIEX, UK), along with XCMS software version 3.2.0 (University of California, Berkeley, CA, USA). The chromatographic conditions were as follows: the column temperature was maintained at 35°C, and the specifications of the chromatographic column were ACQUITY UPLC BEH C18 (100 mm × 2.1 mm, 1.8 µm, Waters, UK), with a flow rate of 0.4 ml/min and an injection volume of 2 µl.

### 2.3 Transcriptome determination and analysis

The early, middle, and late fruit stages of *A. mongolica* were subjected to transcriptome sequencing, utilizing three distinct plants as biological replicates for each group. The extracted RNA underwent purification, followed by library construction, with sequencing libraries generated using the NEBNext^®^ Ultra™ RNA Library Prep Kit (NEB, Ipswich, MA, USA) according to the manufacturer’s instructions. Sequencing was conducted on the Illumina Hiseq platform, with partial execution by Beijing Novozymes Technology Co. The resulting sequencing data were analyzed to evaluate differential gene expression and GO and KEGG enrichment. In the KEGG enrichment analysis, pathways with a *p* ≤ 0.05 were deemed significantly enriched among the DEGs.

### 2.4 Integrated analysis of metabolomics and transcriptomics

The KEGG pathway annotation is conducted on differentially expressed genes and metabolites by employing various gene expression profiles and metabolite concentrations. This approach enables the identification of shared pathways, thereby deepening the understanding of the regulatory mechanisms that influence the growth and development of *A. mongolica* kernels.

### 2.5 Statistical analysis

Data analysis was conducted using SPSS version 17.0 and Excel version 2021. Pairwise comparisons between groups were performed using the t-test. Data are presented as means ± standard deviation (SD), with statistical significance set at *p* < 0.05.

## 3 Results

### 3.1 Comprehensive assessment of metabolomics

**Figure 1:**
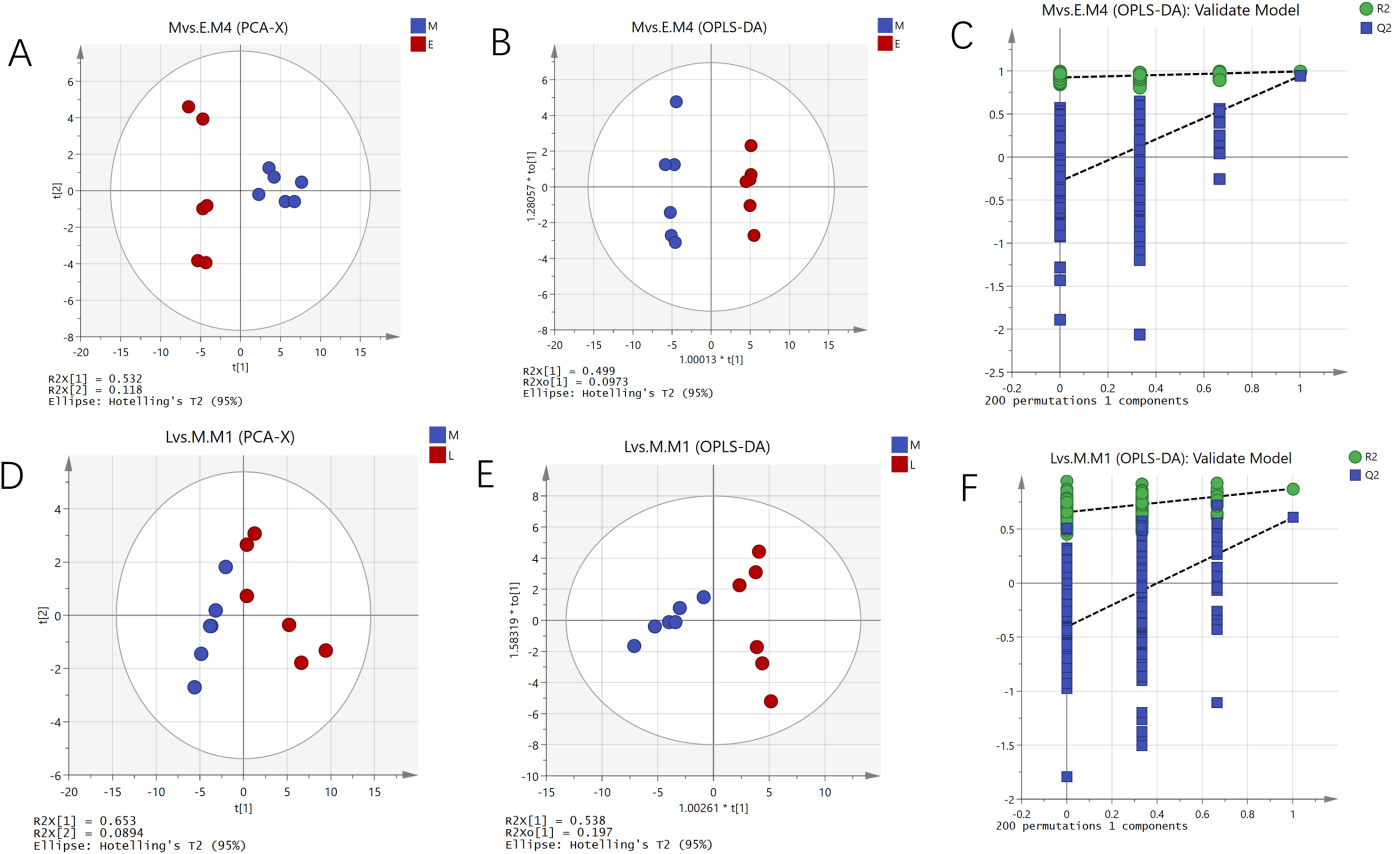
PCA score charts **(A, D)**, OPLS-DA score charts **(B, E)**, and corresponding model validation charts **(C, F)** for group E vs. M, M vs. L.

The results from the PCA of the metabolic profiles comparing groups E and M, as well as groups M and L, indicated a significant differentiation between group E and group M. Between group M and group L, as illustrated in **Figure 1**. This differentiation was supported by the effective clustering of metabolites, which reflects considerable variations in metabolic status among the three groups. Furthermore, OPLS-DA reinforced the significant differences in metabolites between groups E and M and between groups M and L. A permutation test was conducted to assess the potential for overfitting in the OPLS-DA models for these comparisons. The results indicated that all R² and Q² values, moving from left to right, were lower than the original values on the far right, with Q² intersecting the Y-axis in the negative semi-axis. This finding suggests that the model demonstrated strong fit and predictive capability. By applying a threshold of VIP > 1 and a *p* < 0.05 for screening, 139 significant differential metabolites were identified in the comparisons of E vs. M and M vs. L. The variations in the concentrations of these differential metabolites throughout the development of *A. mongolica* are detailed in **Supplementary Tables S1**, **S2**. Specifically, 68 and 36 differential metabolites were identified in the comparisons of E vs. M and M vs. L, respectively. With 29 and 21 metabolites being up-regulated and 39 and 15 metabolites being down-regulated. In our study, the results of the overall metabolic analysis are consistent with the data from some previous studies. In the course of the study, we adopted rigorous experimental methods, so that our data is consistent with the data of other reliable studies, which further proves the credibility of the results of this study.

### 3.2 Analysis of differential abundance and metabolic pathways of metabolites

**Figure 2:**
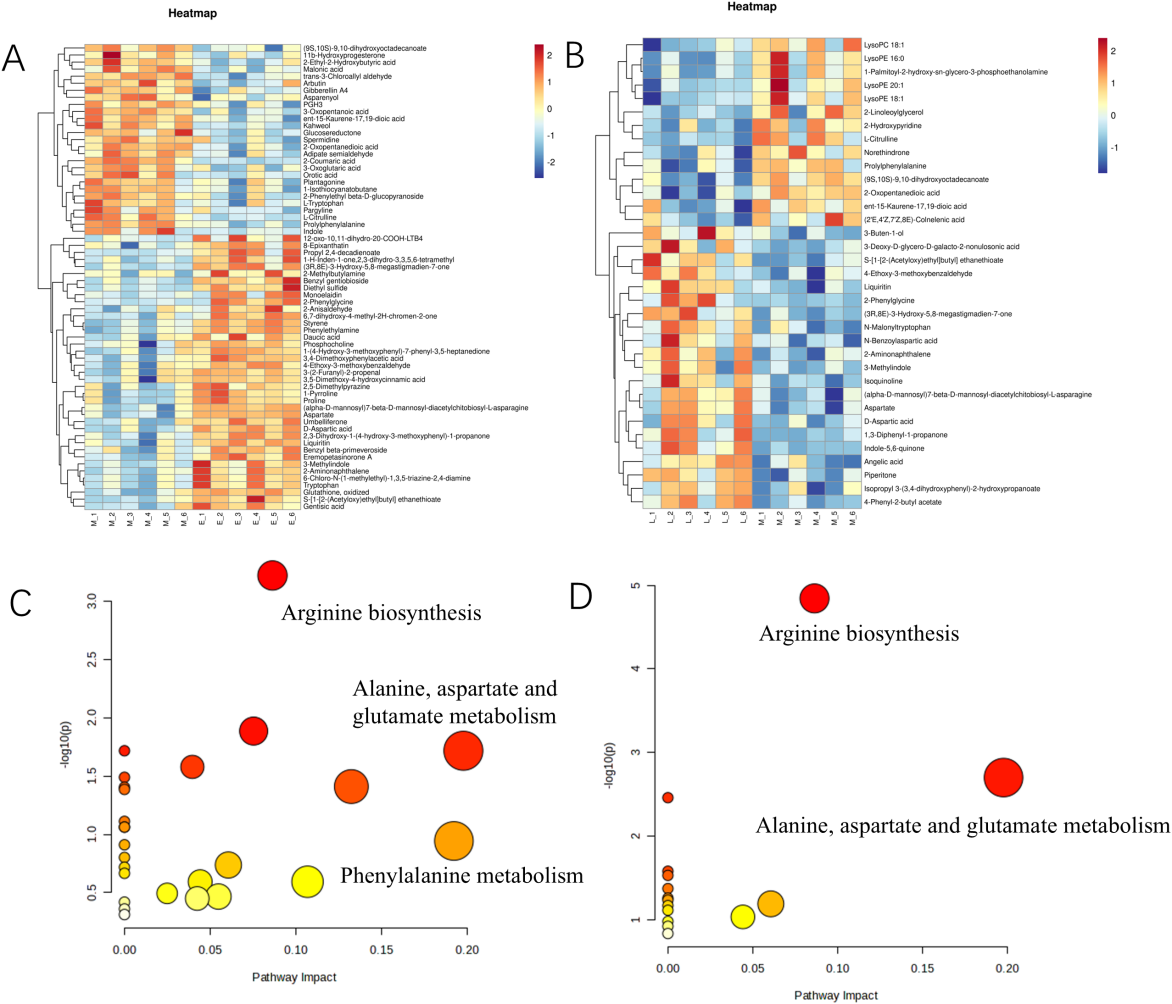
Heat maps of differential metabolites and KEGG enrichment bubble maps of *A*. *mongolica* at different developmental stages. **(A)** E vs. M clustering heat map **(B)** M vs. L clustering heat map **(C)** E vs. M enrichment pathway analysis **(D)** M vs. L enrichment pathway analysis (The horizontal axis represents different periods of the sample, the vertical axis represents differential metabolites, and different colors indicate the colors filled with different values obtained by standardizing different relative contents (red represents high content, blue represents low content). The tree graph on the left side of the heat map represents the hierarchical clustering results of differential metabolites, and the annotation bands on the right side of the clustering graph correspond to substances.).

We found that 88 major differential metabolites were identified in E vs. M and M vs. L. Among them, 52 differential metabolites were identified in E vs. M, with 17 up-regulated and 25 down-regulated. 30 differential metabolites were identified in M vs. L, with 21 up-regulated and 6 down-regulated. Analysis of the results from the early and middle stages revealed that *A. mongolica* was dominated by metabolites such as gentisic acid, phosphorylcholine, malonic acid styrene, and spermidine during the early stages of development. In contrast, succinic acid, orotate, homovanillic acid, and citrulline were increased during the middle stages. The anterior metabolic pathways were mainly focused on amino acid biosynthesis, phytohormone biosynthesis, phenylpropanoid biosynthesis, and spermidine biosynthesis (**Figures 2A, B**; **Supplementary Tables S3**, **S4**). The KEGG and Meta databases were utilized to examine the enrichment of metabolic pathways associated with kernel metabolites at various developmental stages. DEMs identified between the stages E vs. M and M vs. L were mapped to 26 and 15 metabolic pathways, respectively. The principal metabolic pathways pertinent to quality formation include Arginine biosynthesis, beta-alanine metabolism, and Glycerophospholipid metabolism. Notably, the pathways enriched in the E vs. M comparison encompass Alanine, Aspartate, and Glutamate metabolism, Phenylalanine metabolism, and Arginine biosynthesis. Furthermore, the pathways that exhibited significant enrichment in the M vs. L comparison primarily include Arginine biosynthesis, Alanine, Aspartate, and Glutamate metabolism, as well as the TCA cycle, among others. Please refer to **Figures 2C, D** for a visual representation of these findings (**Supplementary Tables S5**, **S6**).

### 3.3 Differential gene expression analysis

**Figure 3:**
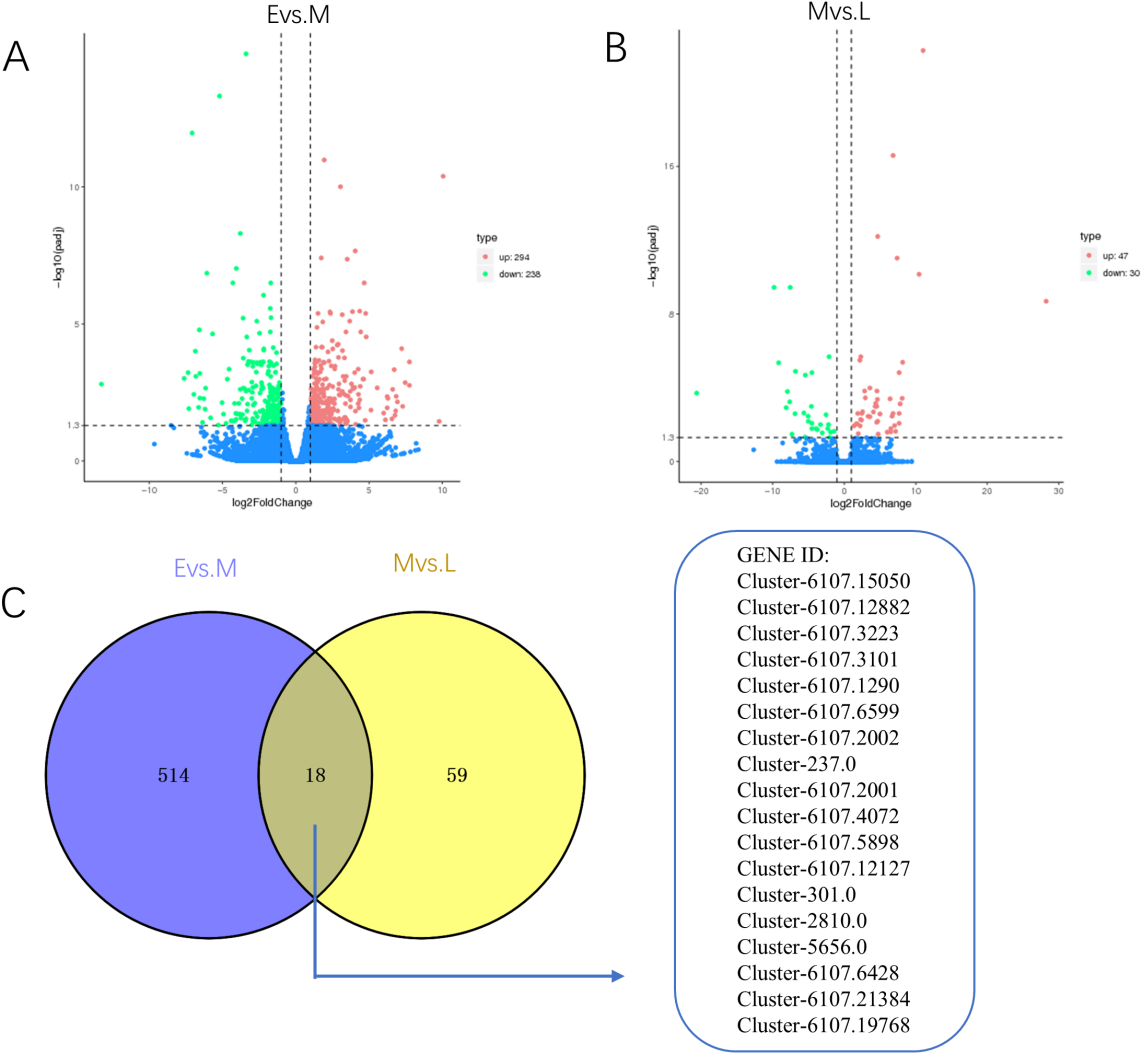
Volcano plots and VEEN plots of differentially expressed genes in *A*. *mongolica* at different developmental stages. **(A)** E vs. M differential gene V-plot **(B)** M vs. L differential gene V-plot **(C)** Veen map of differential genes in different periods.

To elucidate the metabolic alterations occurring in *A. mongolica* throughout its developmental stages, we conducted a transcriptomic analysis aimed at validating gene functionality during these phases. The data derived from the evaluation of gene expression levels underwent a comprehensive examination of global gene differential expression. Utilizing a screening threshold of log2FC ≥ ± 1 and *p* < 0.05, we identified a total of 1,171 DEGs across the three developmental stages. Specifically, between the E vs. M, we identified 532 DEGs, with 294 exhibiting up-regulation and 238 demonstrating down-regulation. Additionally, between M vs. L, we identified 77 DEGs, of which 47 were up-regulated and 30 were down-regulated. Notably, as illustrated in **Figures 3A, B**, 18 DEGs were found to be common to both comparisons (E vs. M and M vs. L), as depicted in **Figure 3C**.

### 3.4 KEGG and GO enrichment analysis of differentially expressed genes

**Figure 4:**
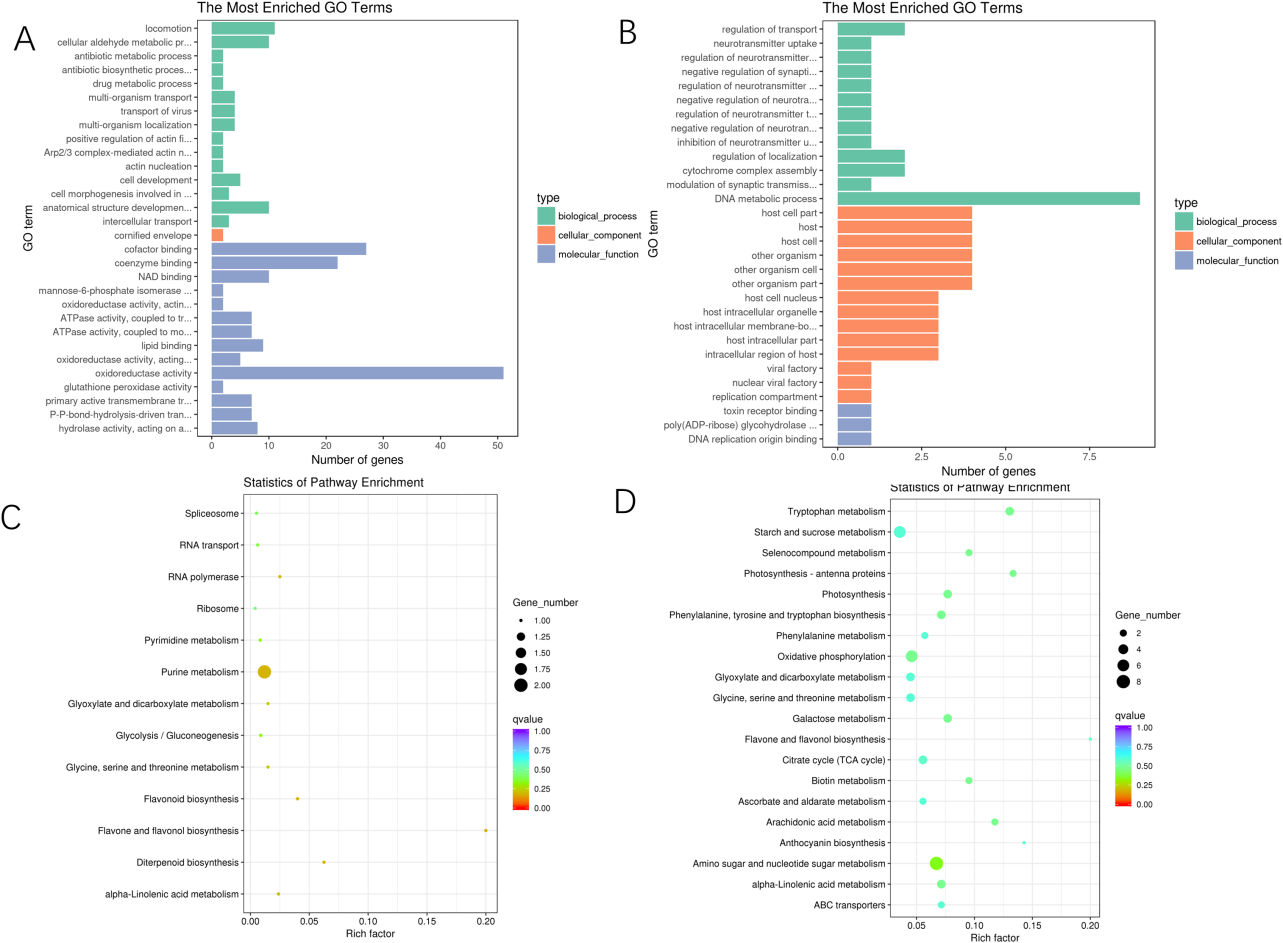
GO enrichment plots and KEGG enrichment bubble plots of differentially expressed genes in *A*. *mongolica* at different developmental stages. **(A)** E vs. M GO database annotation **(B)** M vs. L GO database annotation **(C)** E vs. M KEGG enrichment diagram **(D)** M vs. L KEGG enrichment diagram.

The GO enrichment analysis performed to compare the E and M conditions revealed that, in terms of cellular components, the DEGs were predominantly associated with categories such as cornified envelopes. In the context of molecular functions, the analysis identified significant enrichment in cofactor binding, coenzyme binding, and NAD binding, among other categories. For biological processes, the DEGs showed considerable enrichment in oxidoreductase activity and glutathione peroxidase activity, with a particular focus on oxidoreductase activity. Conversely, the GO enrichment analysis comparing the M and L conditions indicated that, regarding cellular components, the DEGs were primarily enriched in categories such as host cell parts and host intracellular membrane-bounded organelles. The molecular functions were largely concentrated on toxin receptor binding and poly (ADP-ribose) glycopyrrolate activity. Additionally, the biological processes were mainly centered on regulating neurotransmitter levels and the negative regulation of neurotransmitter uptake, among other categories. Please refer to **Figures 4A, B** for a visual representation of these findings (**Supplementary Tables S7**, **S8**).

The KEGG enrichment analysis revealed that 133 differentially expressed genes identified in the comparison between E and M were associated with 62 distinct biological pathways. Notably enriched pathways included those related to amino sugar and nucleotide sugar metabolism, tryptophan metabolism, and photosynthesis - antenna proteins. In the comparison of M vs. L, 14 differentially annotated genes were detected across 13 pathways, with significant enrichment observed in the flavone and flavonoid biosynthesis pathways and the diterpenoid biosynthesis pathway. The pathways associated with flavonoid synthesis and biosynthesis were particularly prominent during the middle and late stages of *A. mongolica* development, suggesting that the accumulation of secondary metabolites, such as flavonoids, primarily occurs during these phases of fruit development. Furthermore, purine metabolism, diterpene biosynthesis, and linolenic acid metabolism exhibited significant activity during the later stages of fruit development, refer to **Figures 4C, D** (**Supplementary Tables S9**, **S10**).

### 3.5 Joint analysis of multi-omics

**Figure 5:**
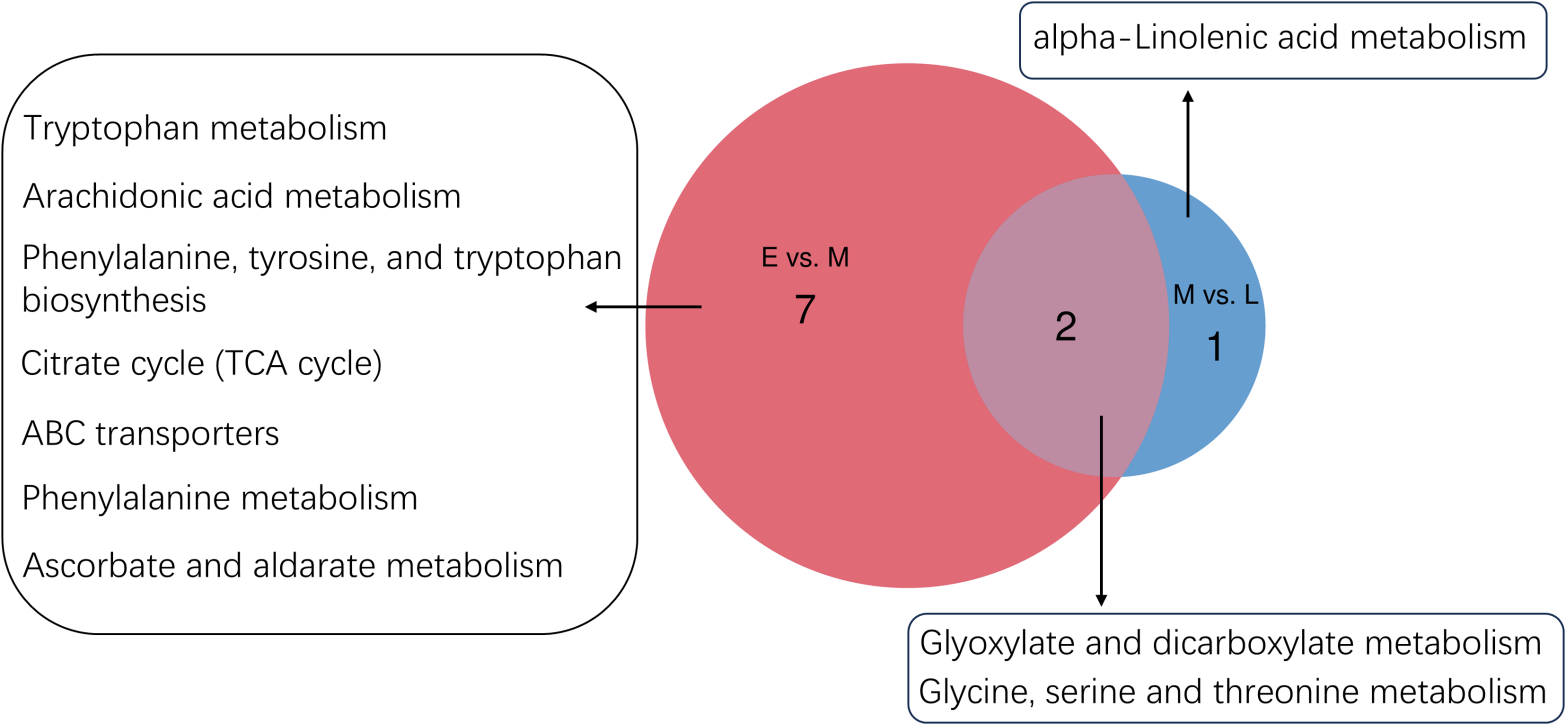
*A. mongolica* developmental E vs. M and M vs. L metabolomics and transcriptomics common pathway.

To investigate the interactions between genes and metabolites during the growth and development of the *A. mongolica* kernel, a thorough analysis integrating transcriptomics and metabolomics was performed on differentially expressed genes and metabolites. During the early and mid-developmental stages of the *A. mongolica* kernel, nine common metabolic pathways were identified, which primarily encompassed phenylalanine metabolism, glycine, serine, and threonine metabolism, tryptophan metabolism, arachidonic acid metabolism, biosynthesis of phenylalanine, tyrosine, and tryptophan, the citric acid cycle, ABC transporters, glyoxylic acid and dicarboxylic acid metabolism, as well as ascorbic acid and aldonic acid metabolism. As the development progressed from mid to late stages, three significantly enriched KEGG pathways were noted: linoleic acid metabolism, glycine, serine, and threonine metabolism, and glyoxylic acid and dicarboxylic acid metabolism, refer to **Figure 5**.

Further examination indicated that during the early and mid-stages, the differential metabolite oxaloacetic acid within the glycine, serine, and threonine metabolism pathways exhibited a downregulation trend, which was associated with a significant downregulation of the differentially expressed genes *LDC*, *gcvP*, *trpA*, *glyA*, and *SHMT*. Concurrently, the *YUCCA* differential gene was upregulated in the tryptophan metabolism pathway. At the same time, *TAA1* was significantly downregulated, resulting in a decrease in the differential metabolite L-tryptophan and an increase in indole. Additionally, in the biosynthesis of phenylalanine, tyrosine, and tryptophan, the *TYRAAT* differential gene was upregulated. In contrast, *PAT*, *AAT*, and *trpA* were downregulated, leading to a notable decrease in the differential metabolite’s indole and L-tryptophan.

In the citric acid cycle, the differential genes *SDHB* and *MDH2* were upregulated, while *DLAT*, *aceF*, and *pdhC* were downregulated, reducing the differential metabolite oxaloacetate. During the transition from mid to late stages in the metabolism of glyoxylic acid and dicarboxylic acid, the differential gene *MDH2* was upregulated, while *GLDC*, *glyA*, and *SHMT* were downregulated, resulting in a significant decrease in the differential metabolite oxaloacetate. In the metabolism of glycine, serine, and threonine, the differential genes *glyA* and *SHMT* were upregulated, while *GLDC*, *gcvP*, and *trpA* were downregulated, leading to a significant downregulation of the differential metabolites L-tryptophan and L-aspartic acid.

## 4 Discussion

Amino acids are essential for the growth and development of plants, as evidenced by empirical research indicating that the amino acid composition in plants varies throughout different growth and developmental stages. In numerous plant species, the concentrations of essential amino acids, such as phenylalanine and glutamic acid, demonstrate a significant positive correlation during the intermediate and later growth phases, which are characterized by a notable accumulation of amino acids and their derivatives during biosynthetic processes. The findings of this study support these observations, revealing that in the early developmental stages of *A. mongolica*, levels of L-tryptophan and L-citrulline were relatively elevated. Conversely, compounds such as (alpha-D-mannosyl) 7-beta-D-mannosyl-diacetylchitobiosyl-L-asparagine, D-aspartic acid, oxidized glutathione, 2-phenylglycine, tryptophan, aspartate, and proline exhibited a decreasing trend. In the intermediate and later stages of development, various amino acids, including 7-beta-D-mannosyl-diacetylchitobiosyl-L-asparagine, N-malonyl tryptophan, N-benzoyl aspartic acid, D-aspartic acid, and 2-phenylglycine, showed an upregulation. The metabolic pathways associated with amino acids during the prometaphase and metaphase stages are primarily enriched in the biosynthesis of phenylalanine, tyrosine, and tryptophan, as well as in the metabolism of alanine, aspartate, glutamate, arginine, proline, glycine, serine, and threonine. The relevant amino acid metabolic pathways during the intermediate and later stages predominantly involve the metabolism of alanine, aspartate, glutamate, cyan amino acids, and lysine biosynthesis. Amino acids are integral to the nutritional composition of fruits and vegetables, playing a crucial role that is intricately linked to carbohydrate metabolism. The concentration and variations of different amino acids within plants, along with the regulation of their transport, are closely linked to the processes of plant growth and development, including primary metabolites such as arginine, leucine, glutamic acid, and tyrosine, as well as polysaccharides and lipids that serve as energy sources for plants (Wang et al., 2024). It has been observed that during the growth and ripening phases of *A. mongolica* fruit, there is a continuous accumulation of most amino acids and their derivatives, with a significant accumulation period occurring from fruit growth to maturity. *A. mongolica* predominantly flourishes in the Gobi Desert region, which enhances its resilience to drought and nutrient-deficient conditions. Amino acids provide a vital nitrogen source for the growth of *A. mongolica*, which is essential for improving the plant’s stress resistance (Wang and Lu, 2020; Zhang, 2023). These amino acids play a vital role in the citric acid cycle and the production of energy. Glutamate undergoes transamination to yield aspartate and alanine, both of which are integral to plant respiration. Aspartate serves as a significant precursor for the citric acid cycle, whereas alanine is converted to pyruvate via transamination, subsequently transforming into acetyl-CoA (Cao et al., 2020; Zou et al., 2023). Consequently, by conducting metabolomic analyses of the diverse metabolites present in *A. mongolica* at various stages of harvesting, one can elucidate the fluctuations in amino acid content during its growth and development. This approach contributes to a deeper understanding of the regulatory mechanisms that influence plant growth and development.

Arginine is recognized as a semi-essential amino acid and is critical as a nitrogen storage nutrient in plants. It acts as a secretagogue, promoting the release of growth hormones such as insulin, thereby fulfilling the nitrogen requirements of plants across diverse developmental stages and environmental contexts (Xiong et al., 2024). For example, in coniferous species, a significant portion of the amino acid profile in stored proteins is attributed to arginine, which contains the highest nitrogen content (Canovas et al., 2007). Arginine is essential for storing and transporting nitrogen within plant systems, with approximately 90% of total free nitrogen in vegetative organs being stored as arginine. Additionally, arginine serves as a precursor in the synthesis of various signaling molecules, including ornithine, proline, and polyamines (Chen et al., 2021). Our research had demonstrated that during the developmental phases of *A. mongolica* (E, M, L), there was a notable and substantial differential metabolic pathway linked to “Arginine biosynthesis.” In this research, we observed that the differential metabolites linked to the arginine enrichment pathway, namely 2-Oxopentanedioic acid and L-Citrulline, were up-regulated during the prometaphase. In contrast, Aspartate demonstrated a transient down-regulation, which was subsequently followed by a notable up-regulation in the later metaphase. On the other hand, both 2-Oxopentanedioic acid and L-Citrulline exhibited a trend towards down-regulation. These findings indicate that arginine plays a vital role in the early and intermediate phases of *A. mongolica* development and growth, thereby supporting the synthesis of essential compounds necessary for the plant’s development.

The phenylalanine metabolism is closely linked to the biosynthesis of various bioactive compounds, including flavonoids and terpenoids, which are synthesized directly or indirectly through the phenylalanine metabolic pathway (Shang and Wu, 2022). Phenylalanine acts as a precursor in the phenylpropanoid metabolic pathway, a vital secondary metabolic pathway in plants that significantly affects growth, development, environmental interactions, and resistance mechanisms to various biological stresses (Dong and Lin, 2021). Phenylalanine plays a significant role in the biosynthesis of lignin via the action of aromatic amino acid deaminase. Its involvement in lignin production is essential for improving plant growth, quality, and resilience to stress, as it contributes to the fortification of the cell wall and the maintenance of structural integrity in plants (Liu et al., 2018). Flavonoids have been successfully isolated and extracted from *A. mongolica* (Zheng et al., 2017), exhibiting therapeutic effects in areas such as anti-tumor (Luiz-Ferreira et al., 2023), anti-hypertension (Haynes et al., 2023), diabetes treatment (Dewi et al., 2023), and organ fibrosis (Dan et al., 2024). The concentration of flavonoids in *A. mongolica* varies across different developmental stages, and the harvest timing can influence the plant’s efficacy. The phenylalanine metabolism is essential for plant growth, development, resilience to adverse conditions, and overall survival (Pascual et al., 2016). Flavonoids significantly enhance photosynthetic efficiency and facilitate the growth and development of plant structures such as roots, stems, and leaves. Additionally, they exhibit a range of biological activities within the organism, including resistance to insect predation, antioxidant effects, and the regulation of endocrine and metabolic functions (Sharma et al., 2019). During the early and middle developmental phases of *A. mongolica*, there was a notable down-regulation of the differential metabolites 2-hydroxycinnamic acid and phenylethylamine, which are integral to the phenylalanine metabolic pathway. This down-regulation was corroborated by the corresponding decrease in the expression of the differentially expressed genes *PAT*, *AAT*, and *4CL*. Additionally, within the biosynthetic pathways of phenylalanine, tyrosine, and tryptophan, the differential metabolites indole and L-tryptophan also exhibited significant down-regulation. In contrast, the gene *TYRAAT* was up-regulated, while the genes *PAT*, *AAT*, and *trpA* were down-regulated. The gene *4CL* demonstrated a decrease in expression during the early and middle developmental stages. Conversely, flavonoid 3’-monooxygenase experienced a significant up-regulation in the pathways related to flavonoid and flavanol synthesis during the middle and late developmental stages. These findings indicate that the accumulation of flavonoids in *A. mongolica* predominantly occurs during the later stages of fruit development.

## 5 Conclusion

This research utilized metabolomic analysis to examine the alterations in various metabolites present in the fruit of *A. mongolica* across different growth stages, while also identifying pertinent genes through transcriptomic validation. The results underscored the significant role of amino acids and their associated metabolic pathways in the plant’s growth and developmental processes. It was determined that amino acid metabolism in *A. mongolica* is intricately linked to the synthesis of flavonoid compounds, with the observed variations at distinct developmental stages being crucial for the plant’s growth and development. Transcriptomic analyses also indicated that the metabolic pathways of differentially enriched genes were primarily associated with phenylpropanoid and flavonoid biosynthesis. The expression levels of these genes exhibited a positive correlation with the plant’s growth processes, reflecting the fluctuations of flavonoid compounds in *A. mongolica*. An examination of the changes in differential metabolites and genes in *A. mongolica* revealed common characteristics within the phenylalanine metabolic pathway. Further investigations affirmed the significance of the arginine and phenylalanine metabolic pathways in the growth and development of *A. mongolica*, thereby offering valuable insights into the regulatory mechanisms that govern plant growth and development. These findings provide a theoretical foundation for further elucidating the biosynthetic mechanisms of plant metabolites and for the cultivation of *A. mongolica*.

## Data Availability

The datasets presented in this study can be found in the National Center for Biotechnology Information Read Archive (SRA) database (https://www.ncbi.nlm.nih.gov/biosample/48483481).
