# Supplementary material for: Metabolomics and transcriptomics indicate the changes in medicinal components of Amygdalus mongolica kernels during different developmental stages
Source: Front Plant Sci. 2025 May 23;16:1597638. doi: 10.3389/fpls.2025.1597638 (PMC12141290; doi:10.3389/fpls.2025.1597638)
Supplement: Supplementary file 2 [file Table1.docx]

Figure S1. *A.mongolica* fruit at different developmental stages (The upper part of the picture shows the flesh of Amygdalus mongolica from various periods, and the lower part shows the inner core of *A. mongolica*)

Table S1. Screening results of S vs. M differential metabolites in *A. mongolica*

| Ion mode | Hmdb | Common name | ratio | p.value | Regulated |
| --- | --- | --- | --- | --- | --- |
| ﹣ | METPA1645 | Gibberellin A4 | 4.61E+00 | 3.78E-03 | **↑** |
| ﹣ | HMDB0060516 | trans-3-Chloroallyl aldehyde | 3.88E+00 | 3.61E-04 | **↑** |
| ﹣ | HMDB0059633 | (9S,10S)-9,10-dihydroxyoctadecanoate | 4.06E+00 | 4.01E-03 | **↑** |
| ﹢ | HMDB0041304 | Asparenyol | 2.08E+00 | 2.16E-02 | **↑** |
| ﹣ | HMDB0040261 | Glucosereductone | 2.10E+00 | 3.12E-03 | **↑** |
| ﹢ | HMDB0036726 | ent-15-Kaurene-17,19-dioic acid | 6.11E+00 | 2.00E-03 | **↑** |
| ﹢ | HMDB0035602 | Kahweol | 4.17E+00 | 4.57E-04 | **↑** |
| ﹢ | HMDB0033482 | Plantagonine | 2.69E+00 | 4.42E-02 | **↑** |
| ﹢ | HMDB0031328 | 1-Isothiocyanatobutane | 3.05E+00 | 1.71E-02 | **↑** |
| ﹣ | HMDB0029943 | Arbutin | 2.35E+00 | 1.09E-02 | **↑** |
| ﹣ | HMDB0029819 | 2-Phenylethyl beta-D-glucopyranoside | 2.88E+00 | 7.58E-03 | **↑** |
| ﹣ | HMDB00208 | 2-Oxopentanedioic acid | 2.68E+00 | 1.47E-03 | **↑** |
| ﹢ | HMDB0015563 | Pargyline | 1.20E+01 | 2.55E-02 | **↑** |
| ﹢ | HMDB0013701 | 3-Oxoglutaric acid | 2.58E+00 | 1.35E-02 | **↑** |
| ﹢ | HMDB0013040 | PGH3 | 4.15E+00 | 9.73E-03 | **↑** |
| ﹣ | HMDB0012882 | Adipate semialdehyde | 2.80E+00 | 5.88E-03 | **↑** |
| ﹢ | HMDB0011179 | Prolylphenylalanine | 7.39E+00 | 8.70E-04 | **↑** |
| ﹣ | HMDB0004031 | 11b-Hydroxyprogesterone | 2.34E+01 | 3.01E-02 | **↑** |
| ﹣ | HMDB0003771 | 3-Oxopentanoic acid | 2.71E+00 | 1.06E-03 | **↑** |
| ﹢ | HMDB0002641 | 2-Coumaric acid | 4.32E+00 | 1.78E-04 | **↑** |
| ﹣ | HMDB0001975 | 2-Ethyl-2-Hydroxybutyric acid | 2.57E+00 | 3.14E-03 | **↑** |
| ﹢ | HMDB0001257 | Spermidine | 4.88E+00 | 1.11E-02 | **↑** |
| ﹢ | HMDB0000929 | L-Tryptophan | 2.37E+00 | 3.66E-02 | **↑** |
| ﹢ | HMDB0000904 | L-Citrulline | 5.53E+01 | 1.05E-03 | **↑** |
| ﹢ | HMDB0000904 | L-Citrulline | 4.69E+00 | 1.26E-02 | **↑** |
| ﹢ | HMDB0000738 | Indole | 3.37E+00 | 2.80E-02 | **↑** |
| ﹣ | HMDB0000691 | Malonic acid | 3.19E+00 | 2.39E-03 | **↑** |
| ﹣ | HMDB0000691 | Malonic acid | 2.07E+00 | 1.48E-02 | **↑** |
| ﹢ | HMDB0000226 | Orotic acid | 2.04E+00 | 2.42E-02 | **↑** |
| ﹢ | HMDB0136755 | 6,7-dihydroxy-4-methyl-2H-chromen-2-one | 2.27E-01 | 1.95E-03 | ↓ |
| ﹣ | HMDB0062251 | (alpha-D-mannosyl)7-beta-D-mannosyl-diacetylchitobiosyl-L-asparagine | 2.98E-01 | 2.64E-03 | ↓ |
| ﹢ | HMDB0059683 | 1-H-Inden-1-one,2,3-dihydro-3,3,5,6-tetramethyl | 4.78E-01 | 2.99E-03 | ↓ |
| ﹢ | HMDB0041802 | 2-Aminonaphthalene | 3.05E-01 | 3.55E-03 | ↓ |
| ﹢ | HMDB0041636 | (3R,8E)-3-Hydroxy-5,8-megastigmadien-7-one | 1.48E-01 | 4.46E-04 | ↓ |
| ﹣ | HMDB0041515 | Benzyl gentiobioside | 2.62E-01 | 8.83E-04 | ↓ |
| ﹢ | HMDB0041286 | 2,3-Dihydroxy-1-(4-hydroxy-3-methoxyphenyl)-1-propanone | 3.13E-01 | 7.82E-04 | ↓ |
| ﹣ | HMDB0041190 | Benzyl beta-primeveroside | 4.40E-01 | 1.06E-02 | ↓ |
| ﹢ | HMDB0037764 | S-[1-[2-(Acetyloxy)ethyl]butyl] ethanethioate | 3.95E-01 | 2.84E-03 | ↓ |
| ﹢ | HMDB0037307 | Propyl 2,4-decadienoate | 2.41E-01 | 8.93E-03 | ↓ |
| ﹢ | HMDB0036473 | 8-Epixanthatin | 4.81E-01 | 1.13E-02 | ↓ |
| ﹢ | HMDB0035289 | 2,5-Dimethylpyrazine | 4.53E-01 | 1.15E-02 | ↓ |
| ﹢ | HMDB0034240 | Styrene | 1.20E-01 | 2.08E-04 | ↓ |
| ﹢ | HMDB0033766 | 2-Anisaldehyde | 4.55E-01 | 3.49E-02 | ↓ |
| ﹢ | HMDB0033710 | 1-(4-Hydroxy-3-methoxyphenyl)-7-phenyl-3,5-heptanedione | 4.35E-01 | 5.64E-03 | ↓ |
| ﹢ | HMDB0033249 | 6-Chloro-N-(1-methylethyl)-1,3,5-triazine-2,4-diamine | 2.60E-01 | 2.79E-03 | ↓ |
| ﹣ | HMDB0032918 | 3-(2-Furanyl)-2-propenal | 3.88E-01 | 2.21E-02 | ↓ |
| ﹣ | HMDB0032616 | 3,5-Dimethoxy-4-hydroxycinnamic acid | 3.17E-01 | 4.94E-02 | ↓ |
| ﹢ | HMDB0032392 | 2-Methylbutylamine | 7.90E-02 | 4.59E-02 | ↓ |
| ﹢ | HMDB0031666 | Diethyl sulfide | 2.01E-01 | 1.33E-03 | ↓ |
| ﹣ | HMDB0031665 | Daucic acid | 4.39E-01 | 1.26E-02 | ↓ |
| ﹣ | HMDB0029865 | Umbelliferone | 2.75E-01 | 4.90E-03 | ↓ |
| ﹢ | HMDB0029685 | 4-Ethoxy-3-methoxybenzaldehyde | 4.45E-01 | 1.36E-02 | ↓ |
| ﹢ | HMDB0029669 | Eremopetasinorone A | 2.45E-01 | 1.64E-03 | ↓ |
| ﹣ | HMDB0029520 | Liquiritin | 7.78E-02 | 2.93E-03 | ↓ |
| ﹣ | HMDB0012549 | 12-oxo-10,11-dihydro-20-COOH-LTB4 | 1.70E-02 | 3.14E-02 | ↓ |
| ﹢ | HMDB0012497 | 1-Pyrroline | 3.71E-01 | 5.81E-03 | ↓ |
| ﹢ | HMDB0012275 | Phenylethylamine | 1.13E-01 | 3.99E-04 | ↓ |
| ﹢ | HMDB0011567 | Monoelaidin | 1.68E-01 | 4.14E-02 | ↓ |
| ﹢ | HMDB0006483 | D-Aspartic acid | 3.02E-01 | 2.64E-04 | ↓ |
| ﹣ | HMDB0003337 | Glutathione, oxidized | 2.37E-01 | 1.62E-03 | ↓ |
| ﹢ | HMDB0002210 | 2-Phenylglycine | 2.25E-01 | 8.60E-03 | ↓ |
| ﹢ | HMDB0001565 | Phosphocholine | 4.56E-01 | 3.64E-02 | ↓ |
| ﹢ | HMDB0000929 | Tryptophan | 2.55E-01 | 2.77E-03 | ↓ |
| ﹢ | HMDB0000466 | 3-Methylindole | 3.40E-01 | 5.74E-03 | ↓ |
| ﹢ | HMDB0000434 | 3,4-Dimethoxyphenylacetic acid | 2.62E-01 | 1.62E-03 | ↓ |
| ﹣ | HMDB0000191 | Aspartate | 2.93E-01 | 1.46E-03 | ↓ |
| ﹢ | HMDB0000162 | Proline | 3.60E-01 | 4.72E-03 | ↓ |
| ﹣ | HMDB0000152 | Gentisic acid | 2.58E-01 | 4.97E-03 | ↓ |

Table S2. Screening results of M vs. B differential metabolites in *A. mongolica*

| Ion mode | Hmdb | Common name | ratio | p.value | Regulated |
| --- | --- | --- | --- | --- | --- |
| ﹣ | HMDB0062251 | (alpha-D-mannosyl)7-beta-D-mannosyl-diacetylchitobiosyl-L-asparagine | 3.11E+00 | 8.05E-03 | ↑ |
| ﹢ | HMDB0041802 | 2-Aminonaphthalene | 2.22E+00 | 2.24E-02 | ↑ |
| ﹣ | HMDB0041756 | Isopropyl 3-(3,4-dihydroxyphenyl)-2-hydroxypropanoate | 3.65E+00 | 2.20E-02 | ↑ |
| ﹢ | HMDB0041636 | (3R,8E)-3-Hydroxy-5,8-megastigmadien-7-one | 4.57E+00 | 3.40E-03 | ↑ |
| ﹢ | HMDB0039500 | N-Malonyltryptophan | 1.02E+01 | 8.40E-03 | ↑ |
| ﹢ | HMDB0037764 | S-[1-[2-(Acetyloxy)ethyl]butyl] ethanethioate | 2.27E+00 | 6.19E-03 | ↑ |
| ﹢ | HMDB0034975 | Piperitone | 2.43E+00 | 1.34E-03 | ↑ |
| ﹣ | HMDB0034251 | N-Benzoylaspartic acid | 1.15E+01 | 2.46E-02 | ↑ |
| ﹢ | HMDB0034244 | Isoquinoline | 2.70E+00 | 1.49E-02 | ↑ |
| ﹢ | HMDB0032041 | 1,3-Diphenyl-1-propanone | 3.32E+00 | 1.37E-02 | ↑ |
| ﹢ | HMDB0031614 | 4-Phenyl-2-butyl acetate | 3.10E+00 | 3.36E-03 | ↑ |
| ﹢ | HMDB0031324 | 3-Buten-1-ol | 2.27E+00 | 2.10E-02 | ↑ |
| ﹢ | HMDB0029685 | 4-Ethoxy-3-methoxybenzaldehyde | 2.69E+00 | 2.25E-02 | ↑ |
| ﹣ | HMDB0029608 | Angelic acid | 2.11E+00 | 8.62E-03 | ↑ |
| ﹣ | HMDB0029520 | Liquiritin | 5.09E+01 | 8.21E-03 | ↑ |
| ﹣ | HMDB0006779 | Indole-5,6-quinone | 8.65E+00 | 6.47E-03 | ↑ |
| ﹢ | HMDB0006483 | D-Aspartic acid | 3.03E+00 | 4.85E-02 | ↑ |
| ﹢ | HMDB0002210 | 2-Phenylglycine | 9.19E+00 | 2.75E-02 | ↑ |
| ﹢ | HMDB0000466 | 3-Methylindole | 2.12E+00 | 4.05E-02 | ↑ |
| ﹣ | HMDB0000425 | 3-Deoxy-D-glycero-D-galacto-2-nonulosonic acid | 2.95E+00 | 3.41E-02 | ↑ |
| ﹣ | HMDB0000191 | Aspartate | 3.00E+00 | 1.05E-02 | ↑ |
| ﹣ | HMDB11482 | LysoPE 20:1 | 2.62E-01 | 2.26E-02 | ↓ |
| ﹣ | HMDB11475 | LysoPE 18:1 | 3.81E-01 | 9.86E-03 | ↓ |
| ﹢ | HMDB11475 | LysoPE 18:1 | 3.94E-01 | 9.96E-03 | ↓ |
| ﹣ | HMDB11473 | LysoPE 16:0 | 4.44E-01 | 1.25E-02 | ↓ |
| ﹢ | HMDB02815 | LysoPC 18:1 | 4.66E-01 | 8.36E-03 | ↓ |
| ﹣ | HMDB0059633 | (9S,10S)-9,10-dihydroxyoctadecanoate | 1.79E-01 | 1.71E-03 | ↓ |
| ﹢ | HMDB0036726 | ent-15-Kaurene-17,19-dioic acid | 4.21E-01 | 3.83E-02 | ↓ |
| ﹢ | HMDB0030996 | (2'E,4'Z,7'Z,8E)-Colnelenic acid | 3.54E-01 | 3.59E-02 | ↓ |
| ﹣ | HMDB00208 | 2-Oxopentanedioic acid | 4.17E-01 | 3.58E-03 | ↓ |
| ﹢ | HMDB0014855 | Norethindrone | 3.05E-01 | 1.50E-02 | ↓ |
| ﹢ | HMDB0013751 | 2-Hydroxypyridine | 4.89E-01 | 1.81E-02 | ↓ |
| ﹢ | HMDB0011538 | 2-Linoleoylglycerol | 4.05E-01 | 3.14E-02 | ↓ |
| ﹢ | HMDB0011503 | 1-Palmitoyl-2-hydroxy-sn-glycero-3-phosphoethanolamine | 4.52E-01 | 1.86E-02 | ↓ |
| ﹢ | HMDB0011179 | Prolylphenylalanine | 2.54E-01 | 1.63E-02 | ↓ |
| ﹢ | HMDB0000904 | L-Citrulline | 5.20E-02 | 9.56E-03 | ↓ |
